# The Moderating Role of Intelligence and Prior Knowledge for the Effectiveness of a Computer-Based Mathematics Intervention in Students with Low Mathematics Performance

**DOI:** 10.3390/jintelligence14030048

**Published:** 2026-03-13

**Authors:** Moritz Herzog, Michael Grosche, Gunnar Bruns, Gino Casale

**Affiliations:** 1 Institute of Mathematics Education, University of Cologne, 50931 Köln, Germany; 2 Institute of Educational Research, University of Wuppertal, 42119 Wuppertal, Germany

**Keywords:** mathematics intervention, mathematics difficulties, singe-case design, computer-based intervention, intelligence, prior knowledge, moderation analysis

## Abstract

The moderation of intervention effects by intelligence and prior knowledge deserves further investigation, because they inform how to design and implement interventions. This study analyzed the moderation of the effectiveness of a computer-based mathematics intervention in 10 primary school students with low mathematics performance and low-to-average intelligence in an ABAB-single-case research design. Prior knowledge and intelligence were assessed before the intervention. The computer-based intervention trained basic numerical skills. Visual inspection of the learning trajectories revealed a broad heterogeneity of effectiveness of the intervention. A hierarchical piecewise regression analysis across all students revealed a significant negative moderation of the intervention effectiveness through intelligence. Whereas prior knowledge did not have a moderating influence, children with higher intelligence showed slower learning rates during the intervention in this specific low-performing sample. One reason for the negative moderation of the intervention effects could be that the intervention trained strategies and skills that more intelligent students had already developed.

## 1. Introduction

Throughout the past decades, an increasing number of intervention studies for school students regarding mathematics has been published. Especially since 2010, Reynvoet et al. (2021) report an exponential increase in mathematics intervention studies. Several meta-analyses have shown that most of the interventions that have been published were effective—in the sense that children who participated in the intervention could increase their mathematical skills compared to a control group (e.g., Nelson & McMaster, 2019; see Reynvoet et al., 2021 for a meta-review).

However, not all mathematics interventions are equally effective, and the response to interventions might vary across individuals (Fuchs et al., 2012). In their meta-review, Reynvoet et al. (2021) found a broad agreement in meta-analyses that interventions focusing on one specific mathematical skill were more effective than general and broad mathematical interventions. Research suggests that matching the contents of an intervention with the specific needs of the students is a crucial criterion for effective interventions (Burns, 2011; Räsänen et al., 2019). When comparing the effectiveness of interventions addressing different mathematical competencies, basic numerical skills (e.g., number comparison, number line estimation, or simple arithmetic) appear to be especially effective. Intervention effects were also bigger when mathematical language was included (Reynvoet et al., 2021). Munez et al. (2022) reported that blended numeracy and working memory interventions were particularly effective for students with mathematical difficulties. This finding emphasizes that mathematical learning can benefit from training general cognitive resources.

Over the last decade, computer-based mathematics interventions have received increasing attention. Computer-based mathematics interventions employ computers or tablets to foster mathematical knowledge (Räsänen et al., 2019). In general, computer-based interventions can help improve students’ mathematical performance (Byun & Joung, 2018; Ran et al., 2021). Kanive et al. (2014) reported similarities regarding the effects between computer-based and non-digitalized interventions. However, Li and Ma (2010) reported in their meta-analysis of the learning effects of computer-based mathematics instruction that the strongest effects were found for the group of students with special educational needs. Like traditional mathematics interventions, computer-based interventions are especially effective in primary school and regarding basic numerical contents (Räsänen et al., 2019). One possible reason for higher effectiveness in primary school could be a better fit between intervention contents and difficulties of the students. Possible motivational effects of computer-based interventions are often discussed. However, empirical studies hardly provide evidence for this claim (Higgins et al., 2019; Wouters et al., 2013). As they require less teacher resources than traditional trainings, computer-based mathematics interventions can be an effective way to reduce learning gaps after the COVID-19 pandemic (Ökördi & Molnár, 2022).

As not all students respond to mathematics interventions similarly, the moderators for the effectiveness of mathematics interventions are of great interest (Powell et al., 2017). Typically, the target group of mathematics interventions are students with low mathematics performance who lack domain-specific knowledge on the one hand (which constitutes their mathematical problems), and on the other hand, often score lower on intelligence tests (e.g., Stern, 2015). Therefore, intelligence as a general predictor and prior knowledge as a domain-specific predictor for the effectiveness of a mathematics intervention need to be considered (Stern, 2015). Applying a single-case research design offers the chance to trace the learning progress of individual students and to find tentative but very detailed evidence regarding the moderating role of (low) intelligence and prior knowledge for the effectiveness of a math intervention.

### 1.1. Structures of Intelligence and Mathematical Knowledge

One definition of intelligence is the “ability to understand complex ideas, to adapt effectively to the environment, to learn from experience, to engage in various forms of reasoning, to overcome obstacles by taking thought” (Neisser et al., 1996, p. 77).

A widely recognized theory of intelligence is the Cattell–Horn–Carroll Model (CHC-Model) that defines three dimensions (“three-stratum model”) of intelligence: A general factor (*g*) covers a wide range of cognitive abilities (first dimension). The general factor is constituted of several subcomponents (second dimension) that define broad abilities. The broad abilities can be further specified into narrow abilities (third dimension) (Schneider & McGrew, 2018). The most prominent broad abilities are gained knowledge (“crystalized intelligence”, *Gc*) and general reasoning skills (“fluid intelligence”, *Gf*). Whereas the heritability of *Gf* is quite large, *Gc* is strongly tied to environmental factors such as socioeconomic status, learning opportunities, education, or cultural background (Ortiz, 2015). However, genetic and neurological entities (such as the nervous system) always need positive environmental conditions to develop (e.g., Gabrieli et al., 2015).

Regarding the internal structure of mathematics, hierarchy does not refer to dimensions of abilities, but to their temporal development: More complex contents build up on simpler contents (Räsänen et al., 2019). For instance, children need to develop counting skills before they can establish size relations of numbers and reliably identify the greater number when presented with two numbers (Le Corre, 2014). Based on the empirically, as well as theoretically, underpinned hierarchy of mathematical content, competence models of mathematical learning have been developed (Fischer et al., 2017). Thus, for a mathematical skill B, there exists a mathematical skill A that needs to be mastered before learning B: in other words, A is the prior knowledge of B.

Based on this understanding, prior knowledge goes beyond a predictor variable. A predictor can estimate later mathematical achievement to some extent, but does not need to be developmentally related. For example, rapid automatized naming of colors is a stable predictor for mathematical performance (Koponen et al., 2017); however, naming colors as fast as possible can hardly be claimed to be an immediate cognitive basis for mathematical learning. In contrast, counting skills might facilitate children’s understanding of number size relations by linking quantities to the order of number words (Le Corre, 2014). In the context of intervention studies, prior knowledge is usually identified with the performance in the pre-tests.

### 1.2. Predicting Children’s Mathematic Achievement

Several studies have investigated the influence of intelligence as well as prior knowledge on students’ mathematical learning. Chu et al. (2016) followed kindergarteners over three years. Mathematical performance at the beginning of schooling was significantly predicted by initial numerical competencies such as counting and quantity comparison. However, intelligence (measured with a Wechsler scale) and executive functions were no significant predictors of mathematical performance at the beginning of schooling. This is in line with Stern (2015, p. 327), who emphasized the “pivotal role of prior knowledge for further learning and advanced performance”.

Prior knowledge is the strongest predictor for mathematical learning; however, intelligence still plays a unique role for mathematical learning (Geary et al., 2020; Stern, 2015; Taub et al., 2008). Geary et al. (2020) reported a correlation of *r* = 0.60 between IQ (*g*) in Grade 7 and mathematical performance in Grade 8. In line with these results, Taub et al. (2008) found strong direct effects of general intelligence (*g*) on mathematical performance in a structural equation model between *r* = 0.68 and 0.85 in several age groups ranging from 5 to 19 years. In general, the direct and indirect effects of intelligence and its subcomponents were stronger in younger students than in older students (Taub et al., 2008). In sum, intelligence is a good predictor for initial performance at the beginning of school, but prior knowledge predicts knowledge gains better (Geary et al., 2017; Renkl & Stern, 1994). 

The interplay of intelligence and prior knowledge appears complex. In a longitudinal study, Kucian (2021) compared the predictive power of intelligence and numeracy in kindergarten on second grade calculation performance. Numeracy predicted subsequent calculation far better than intelligence. However, in kindergarten and first grade, intelligence and numeracy correlated substantially and was stronger than working memory and numeracy. In a recent study, Fiedler et al. (2025) investigated the influence of cognitive abilities and prior knowledge on mathematics achievement from first to fourth grade. In general, cognitive abilities and prior knowledge predicted mathematics achievement in the subsequent years; however, prior knowledge was the stronger predictor in most cases.

### 1.3. Moderator Effects on Mathematics Interventions

Whereas longitudinal studies are important to identify predictive variables, only intervention studies with a pre–post design can give insight into the effectiveness of certain teaching methods for students with different profiles of intelligence or prior knowledge. In this regard, moderator variables play an important role, because interventions tend to not be generally effective, but their effectiveness might depend on additional variables. For example, Hassler Hallstedt et al. (2018) trained primary school students’ simple addition and subtraction skills in a computer-based intervention for three months. Students’ mean arithmetic fluency increased significantly directly after the intervention as well as in six- and twelve month delayed follow-ups. The effectiveness of the intervention was moderated by intelligence: Students with lower IQ scores benefited more from the intervention than students with higher IQ scores. Hassler Hallstedt et al. (2018) suggest compensation effects as one reason for the moderating effects of IQ: Students with low cognitive resources might benefit more from an intensive intervention, because they are provided with necessary resources (e.g., strategies).

Thurn et al. (2022) investigated the influence of intelligence on the effects of an intervention on proportional reasoning in physics. The authors did not only find a positive direct effect of intelligence on the intervention outcomes, but also found that mathematical prior knowledge significantly mediated the influence of intelligence on the effectiveness of the intervention. This finding corroborates the impact of intelligence on mathematical skills on the one hand, and the importance of a differentiated investigation of the effects of intelligence and mathematical skills (in terms of prior knowledge) on subsequent mathematical performance.

Powell et al. (2017) showed that processing speed and reasoning did not significantly moderate the effects of interventions on calculation, nor on word-problem solving. These findings are in line with Swanson et al. (2013), who did not find moderator effects of fluid intelligence on a guided-instruction strategy intervention regarding simple arithmetic. However, Swanson et al. (2013) reported that prior knowledge substantially moderated the effects of a word-problem intervention in favor of more proficient students. That means that students with more domain-specific knowledge had bigger performance gains than students with lower prior knowledge. Similar findings were reported in a game-based intervention for early numeracy in kindergarteners and first-graders by Herzog and Fritz (2022). Children with higher prior knowledge benefited strongly from the intervention. However, classification and matrices as subcomponents of (fluid) intelligence did not moderate the intervention effects.

Cases in which a higher prior knowledge was associated with higher effects of instruction or intervention are often referred to as the “Matthew-effect” (Stanovich, 1986). This effect assumes that higher prior knowledge forms a basis on which new knowledge can be more effectively acquired. Simonsmeier et al. (2021) summarize five reasons for the Matthew-effect: Higher prior knowledge (1) might drive students’ attention to new learning opportunities; (2) helps them to derive new information; (3) facilitates the chunking of new information, which makes it easier to store and retrieve from the long-term memory; (4) usually goes along with more efficient strategies for learning; (5) supports students in finding reliable sources for new information.

However, performance gaps might also close over time, the so-called “compensation effect”. Following Simonsmeier et al. (2021), at least five reasons are accountable in this regard: Higher prior knowledge (1) might lead to fast and thus incorrect or incomplete conclusions without evaluation; (2) might drive attention only selectively to information fitting to the prior knowledge; (3) could be associated with correct routines that hamper the development of new, more efficient strategies; (4) increases the risk that information is mixed up within a specific domain; (5) might interfere with new information that is hard to integrate in the prior knowledge.

In a recent meta-analysis, Simonsmeier et al. (2021) did not find clear evidence in favor of or contradicting the Matthew-effect (nor the compensation effect). Thus, high prior knowledge is not necessarily associated with bigger or smaller intervention effects. The authors underline the need to pay attention to the conditions under which prior knowledge effects interventions positively.

### 1.4. The Current Study

In sum, several studies suggest that students with higher prior knowledge tend to benefit more from teaching and interventions. Intelligence shows similar effects only at a young age and in a smaller degree. However, longitudinal predictor studies can only limitedly explain how intelligence and prior knowledge affect mathematical learning. Intervention studies with a pre–post design can provide more information in this regard, as they control students’ learning opportunities and processes. This allows us to identify moderator variables for mathematical interventions. However, pre–post-designed intervention studies can only provide information about how intelligence and prior knowledge affect intervention outcomes afterwards—information about how intelligence and prior knowledge moderate intervention effects *during* the intervention is thus missing. Information regarding the processes involved during interventions would not only explain why some students benefit more from teaching, but also inform us about the factors that affect interventions. In addition, pre–post designs usually measure the effectiveness of an intervention on a group level (e.g., mean scores). However, information on individual differences regarding the effectiveness of an intervention is often lost in pre–post designs. Information on individual responsivity to an intervention can help to understand and improve the effectiveness of mathematics interventions. Single-case design studies can provide such information, as they track students’ learning outcomes over time with a progression-based assessment (Maggin et al., 2018).

To the best of our knowledge, there are no single-case design studies on the influence of intelligence and prior knowledge on the effectiveness of mathematics interventions. Therefore, the current study can give valuable information regarding the effectiveness of mathematical interventions on an individual level. This information might be useful when designing or choosing appropriate interventions for students with mathematics difficulties. By adapting the research design from a group level (e.g., pre–post design) to an individual level (e.g., single-case design), intervention effects and how they are moderated by intelligence and prior knowledge can be investigated more individually. In intervention studies with a pre–post design on a group level, intelligence and prior knowledge usually are added as covariate variables to control the performance of the outcome measures for the initial performance. By estimating the interaction between intervention effects and prior knowledge or intelligence, the influence of both individual variables on the intervention effects can be investigated.

The aim of the current study was to investigate the moderating effects of intelligence and prior knowledge as general and domain-specific cognitive moderators on a computer-based mathematics intervention. To evaluate the onset of possible intervention effects as well as effects on level or slope, a multiple baseline single-case design was employed. The current study was guided by the following research questions:

1. Does a computer-based mathematics intervention effectively improve mathematics performance in students with low mathematic achievement?
2. How do intelligence and prior knowledge moderate the intervention effects in regard of level and slope?

For both research questions, we hypothesize that students acquire mathematic skills (H1) and maintain these skills (H2) after the intervention ended. For the second research question, we expect moderation effects for both intelligence and prior knowledge for acquisition (H3) and maintenance (H4).

## 2. Materials and Methods

### 2.1. Sample

In total, N = 10 students from three German primary schools took part in this study. Six students were in Grade 2 and four students were in Grade 4. With three exceptions, all students were monolingual German speakers. Of the three multilingual students, one (Bert) spoke Hausa as their first, and German as their second, language, whereas two students (David, Eric) spoke German and Italian as first languages. All students spoke German good enough to follow the instructions of the tests and the intervention. Two students attended a school for special educational needs and the other students attended regular primary schools, located in similar urban areas from three cities in a western part of Germany.

The aim of the study was to investigate the effectiveness of an intervention for students with low mathematical achievement. Following Cirino et al. (2015), performance below the 25th percentile (equals a T-value of 43) served as inclusion criterion. Students were selected from Grades 2 to 4 to cover the whole age range of the intervention. As the intervention addresses students with mathematical learning difficulties, regular primary schools, which are nearly all inclusive in the western parts of Germany, as well as special educational needs schools were included. A power test indicated that a sample size of at least nine cases would be sufficient for the intended analyses, particularly the hierarchical piecewise regression models. However, after consideration with the schools, we included all relevant cases based on the screening.

In advance of the intervention, all students (N = 68) of the participating classes were screened for low mathematical performance (T ≤ 43) with a standardized math test (HRT; see below). Based on the screening results, ten students with low mathematical performance were included in the study. All students meeting the inclusion criteria were asked to participate; parental consent was gained from the ten participating students. Students’ performance in a non-verbal intelligence test (CFT 1-R or CFT 20-R; see below) was average-to-low (T-values ranging from 30 to 46). Table 1 provides an overview of the participants. Unlike intelligence, low mathematical achievement was an inclusion criterion for the intervention.

Written consent was obtained from the parents in advance. The local ethics committee of the University of Wuppertal approved the study.

### 2.2. Instructors

Data collection and intervention was conducted by three trained university students at the end of their bachelor studies in special education. During the last two semesters before the study, all instructors had successfully completed several courses on diagnostics and intervention strategies with special regard to mathematics. During the intervention, the instructors have been closely supervised by the authors.

### 2.3. Instruments

#### 2.3.1. Math Achievement

Students’ mathematical achievement was assessed with the arithmetic subscale of the Heidelberger Rechentest 1–4 (HRT; Haffner et al., 2005). The HRT is a standardized arithmetic test normed for students from Grade 1 to Grade 4. The arithmetic subscale of the HRT covers addition, subtraction, multiplication, division, complement tasks and number comparison in a timed format. In Grade 2, multiplication and division are omitted, because these operations are not yet covered by the curriculum. For each operation, students have two minutes to solve as many of a total of 40 tasks as possible. Thus, a maximum of 240 points was possible. The HRT showed a sufficient retest reliability in the norming study (*r*_tt_ = 0.77–0.89).

#### 2.3.2. Mathematical Prior Knowledge

Students’ domain specific prior knowledge was assessed with the computer-based test CODY-M 2-4 (Kuhn et al., 2018). The CODY-M 2–4 is a standardized and normed mathematics test for students from Grade 2 to Grade 4. Based on a robust indicator approach, the CODY-M 2–4 covers counting skills, simple arithmetic, number line estimation, quantity comparison, transcoding skills, and visual working memory. The number of items per subtest differs between 6 (matrix span) and 48 (quantity comparison). The retest reliability of the CODY-M 2–4 in the norming study was *r*_tt_ = 0.88.

The CODY-M 2–4 was chosen to measure domain-specific prior knowledge for two reasons. First, the test covers a broad set of early numerical skills. Second, the CODY-M 2–4 was designed to match the intervention. Thus, the CODY-M 2–4 provides a holistic and specific measure of the students’ prior mathematical knowledge that is focused during the intervention.

#### 2.3.3. Mathematical Learning Progression

The mathematical development was assessed with the progression-based assessment of the computer intervention CODY-LM (Schwenk et al., 2017). The CODY-LM is a short computer-based test covering addition, subtraction and number ordering in a timed format. Depending on the reaction time, students can gain virtual coins for correct answers and loose virtual coins for wrong answers. The CODY-LM takes five minutes. Depending on the students’ response times, the number of items varies. Across the total intervention time of three months, we administered between 20 and 26 tests for each individual student in order to model their learning progression. Schwenk et al. (2017) reported that the Cody-LM has a good split-half reliability (*r*_split-half_ = 0.87–0.93).

#### 2.3.4. Intelligence

Students’ cognitive ability was assessed with the culture-fair intelligence test CFT 1-R (Weiß & Osterland, 2012) in Grade 2 and CFT 20-R (Weiß, 2006) in Grade 4. Different intelligence tests were used, as the norms (T-values) were not available for the whole age group in one of the tests. T-values in both tests compare the individual performance with a representative norming sample. Both tests are consistent with the CHC model and focus on fluid intelligence (*Gf*). Both versions cover seriation, classification, and matrices. The CFT 20-R additionally covers topological conclusions. For each subtest, students have three to four minutes of time to complete as many of 15 items as possible. Thus, a maximum of 45 (CFT 1-R), respectively 60 (CFT 20-R), was possible. Both tests are normed for the corresponding age group. Both versions of the CFT showed sufficient retest reliabilities in the norming studies (CFT 1-R: *r*_tt_ = 0.88–0.95; CFT 20-R: *r*_tt_ = 0.80–0.82).

### 2.4. Intervention

Students were trained with the computer-based mathematic intervention Meister Cody (“Master Cody”, Kaasa Health, 2013). Meister Cody focusses on so-called robust indicators, i.e., mathematical subskills that predict mathematical achievement well. Typical intervention contents were counting skills, number line estimation, quantity comparison, or simple arithmetic problems. Task formats covered timed tasks (e.g., comparing quantities approximately as fast as possible, including subitizing and groupitizing; Starkey & McCandliss, 2014), visualizations of simple addition and subtractions tasks (e.g., using a twenty-frame, Obersteiner et al., 2014), and arithmetic strategies (e.g., applying decomposition strategies, Laski et al., 2014). Thus, the intervention covers both preschool as well as primary school mathematics. The intervention has a continuous story line with several protagonists. One of the protagonists serves as guide and supports during tasks through hints.

Based on the results of the CODY-M 2–4, Meister Cody adapts the training sessions to the individual prior knowledge as well as the individual progression during the training sessions. The effectiveness of Meister Cody was evaluated empirically in a control group setting (Kuhn & Holling, 2014). In a controlled single-case study with 11 primary school students (Herzog & Casale, 2022), the students’ responsiveness to Meister Cody was mixed underscoring the importance of further examinations focusing on individual patterns of the effectiveness and on moderating variables.

The intervention was conducted on a tablet in a quiet and separate room in the schools during math lessons. One training session was about 20 min. The students received between 10 and 12 training sessions (M = 11.20, SD = 0.87; see Table 1) in total over six weeks, split into two intervention phases (see design). Training sessions were planned twice per week, thus 12 sessions were planned. According to Svane et al. (2023), mathematics interventions typically last between one and six months. Thus, the duration of the current study was aimed at this range. During the sessions, the children do several short exercises on different basic numerical skills. An ongoing storyline provides a coherence between the different exercises. Due to the high degree of standardization, we assumed that the implementation fidelity was comparably high. Whereas the intervention was computerized, setting and implementation fidelity were controlled by the student instructors. All exercises and explanations were given verbally to the students by the computer program. The student instructors provided no additional explanations or hints, but made sure that the intervention program was implemented adequately. As the instructors also supervised the initial and monitoring assessments, the performance of the students was not blinded to them.

### 2.5. Design

Compared to control group intervention studies, a controlled single-case design has several advantages. First, a single-case design allows us to include participants’ characteristics as explaining variables for intervention success (Riley-Tillman et al., 2020). Second, the longitudinal observation of students’ learning progression not only allows us to investigate general intervention effects and moderators based on the learning outcomes, but also to differentiate between level and slope effects (Huitema & McKean, 2000). Third, a single-case design including a maintenance phase (e.g., second baseline phase) makes it possible to investigate how newly learned knowledge is maintained over time after the intervention and which moderating factors might affect the maintenance of new knowledge (Horner et al., 2005). In particular, the comparison of two baseline phases with an intervention phase in between allows us to detect possible transfer effects of the intervention that set in after the intervention ends.

**Figure 1:**
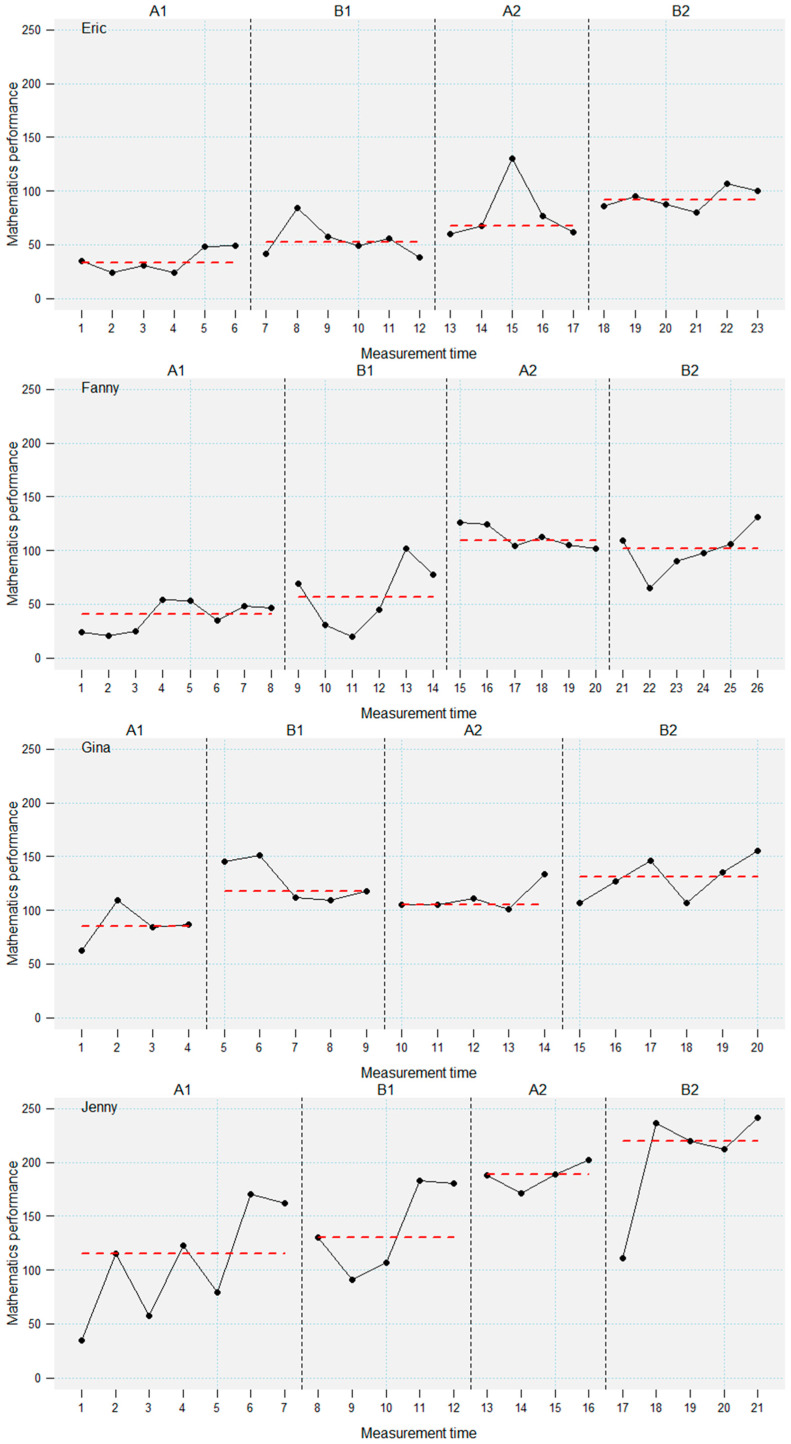
Performance trajectories of the students who benefited in all phases. Dashed lines denote phase medians.

**Figure 2:**
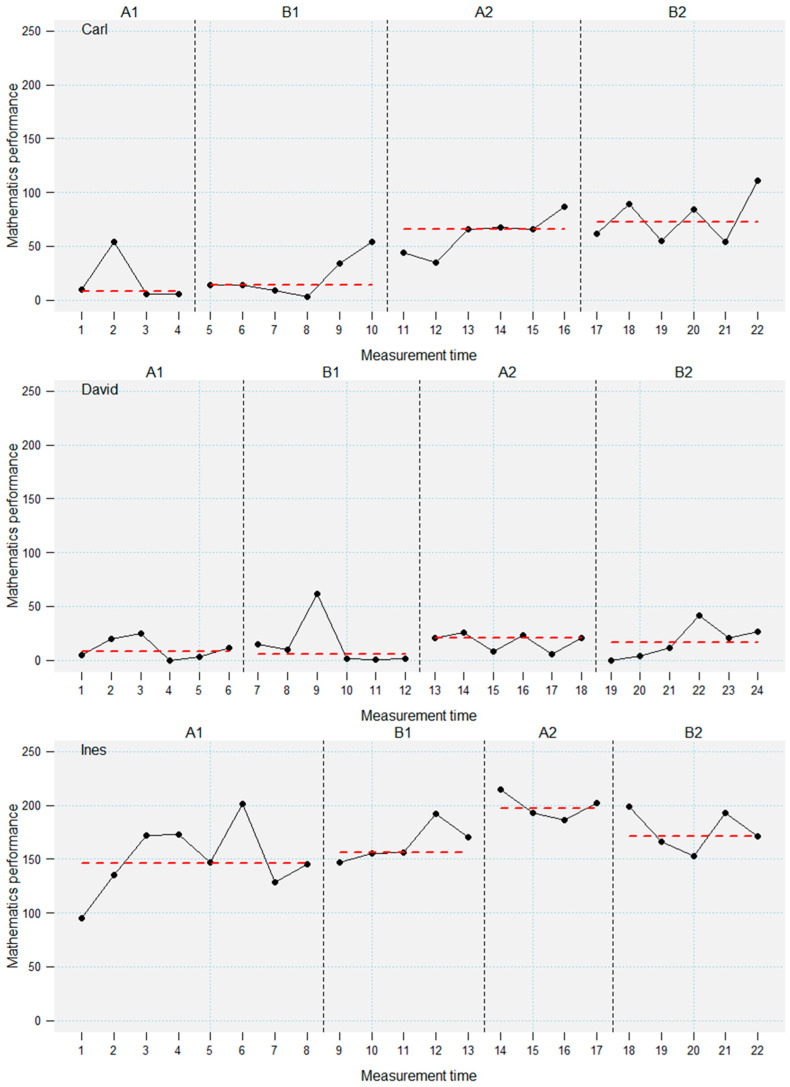
Performance trajectories of the students who benefited in the second A-phase. Dashed lines denote phase medians.

**Figure 3:**
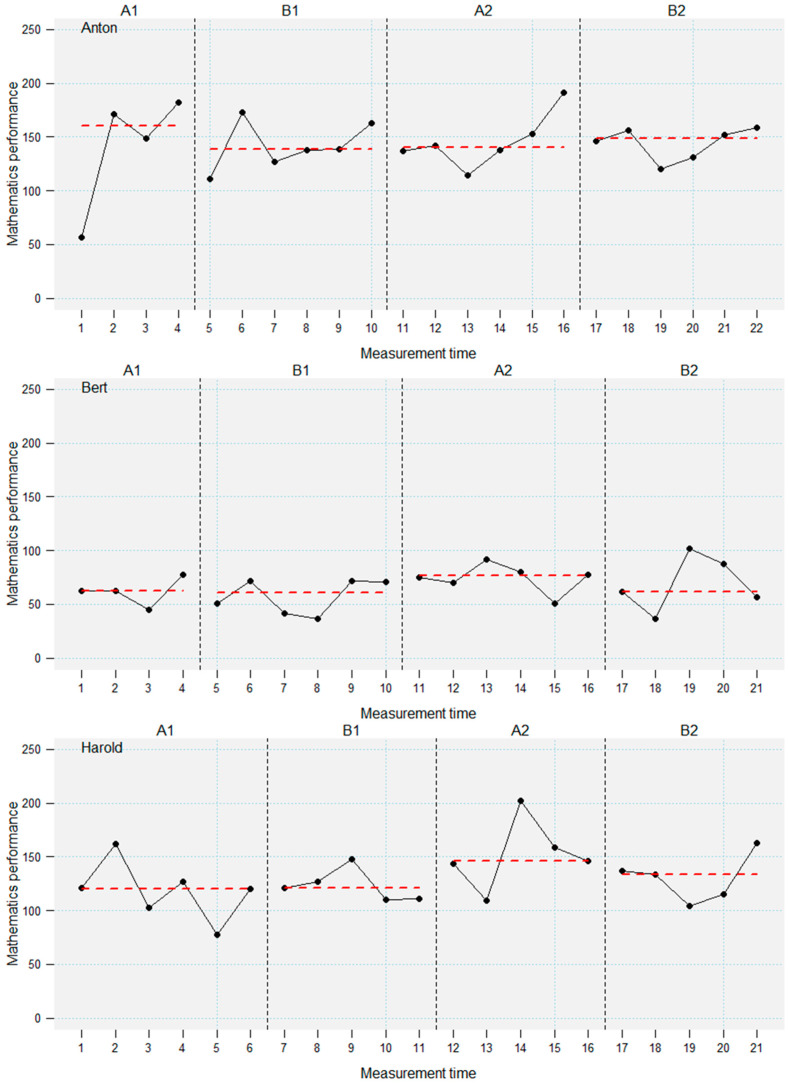
Performance trajectories of the students who did not benefit from the intervention. Dashed lines denote phase medians.

For these reasons, a quasi-experimental controlled single-case ABAB-design with multiple baselines across several participants was employed. During the whole study period, the assessment of students’ mathematical performance with the CODY-LM assessment was planned twice per week. Unplannable events (e.g., illness) led to small variance in measurement time points (see Figure 1, Figure 2 and Figure 3). Students were observed in a baseline phase (A1), followed by an intervention phase (B1). After the first intervention, a second baseline phase (A2, “maintenance”) and a second intervention phase (B2) were conducted. Two baseline and intervention phases were included to investigate the effect of prior knowledge and intelligence on acquisition and maintenance of new mathematical knowledge. Phases A1, B2, and A2 lasted three weeks for each student, including six planned measurement time points per phase. The design included a multiple baseline, which means that the length of the first baseline phase (A1) was randomly varied between two and four weeks, leading to four to eight measurement time points. The onset of the intervention (B1) was staggered across the students to control for external influences (e.g., curriculum) and increase internal validity (i.e., making intervention effects more likely to be ascribed to the intervention; Horner et al., 2005; Kratochwill & Levin, 2010). The intervention was conducted alongside the general mathematics classes. That means that during all phases, students received the usual mathematics teaching by their teachers.

The analyses were planned in two steps. First, a visual inspection of the trajectories of the mathematical learning progression over time was employed to investigate the general effectiveness of the intervention for the students. Second, hierarchical piecewise regression models with the scan R-package (version 0.67; Wilbert & Lüke, 2021) were run across the repeated measurements of all ten cases, as they offer more precise information than standard overlapping indices. In these models, the mathematical performance at the different measurement time points is predicted by the phase (indicating a level effect between the compared phases), the measurement time point within the B-phase (indicating a slope effect), and the measurement time point in general (to control for a trend effect in the data), as well as different individual characteristics such as intelligence, prior knowledge, and possible interactions between these variables. Note that *scan* does not control autocorrelation. For the first research question, neither intelligence nor prior knowledge were entered into the regression equation, testing the intervention effects in general to statistically corroborate the visual inspection results. For the second research question, intelligence and prior knowledge and their interactions were included in order to test moderation effects.

## 3. Results

### 3.1. Visual Inspection

Visual inspection of the plotted learning progressions in Figure 1, Figure 2 and Figure 3 reveals different intervention effects in different students. We grouped the effects in three categories. Out of 10 students, 4 students showed performance gains in all phases (see Figure 1), which indicates both immediate level and slope effects. However, there was considerable variance in the intervention effects in these students. Especially three students (Eric, Fanny, and Jenny) showed only slightly immediate intervention effects. Interestingly, these three students showed a substantial increase in performance during the second A-phase, indicating a delayed intervention effect. All of the students in this group had T-values of at least 40 in prior knowledge, whereas intelligence ranged between 30 and 46.

Another three students showed increased performance in the second A- and B-phase, but not in the first B-phase (see Figure 2). This indicates a delay in the intervention effects in these students. Moreover, these students show only level, but no slope, effects. These students vastly differed in prior knowledge (T-value range 24–45) and intelligence (T-value range 30–41).

Finally, three students showed no considerable intervention effects (see Figure 3). Obviously, these students did not benefit noticeably from the intervention. This group showed relatively high intelligence (T-value range 39–45), but differed in prior knowledge (T-value range 29–50). Both students who visited schools for children with special educational needs are in this group of non-responders.

The findings of the visual inspection were supported by the descriptive statistics of the progression-based assessment across the four phases (see Table 2). Effect sizes for performance differences between the phases (Hedge’s *g*) further corroborate the results of the visual inspection. The large variance in performance gains in the learning progression has to be highlighted. These results indicate an intervention effect that ranges from minimal effects to substantial performance increases in different students.

### 3.2. Overall Effectiveness of the Mathematic Intervention (Research Question 1)

Intervention effectiveness was tested with single-case design specific measures, including overlap indices and randomization-based tests. Overlap indices covered PEM (percentage exceeding the median) and PAND (percentage of all non-overlapping data), which allow estimation of the effect size of the intervention. Whereas the PEM indicates central tendencies in performance across the phases, the PAND gives information on the differences in the performance ranges across the phases. Kendall’s Tau-U with baseline correction and randomization tests provide further effect measure and particularly statistical inference for intervention effects. Tau-U quantifies the magnitude of change between phases (optionally controlling for baseline trend), whereas randomization tests assess whether the observed effect is unlikely under the set of admissible phase randomizations defined by the design.

Overlap indices and randomization-based tests were in line with the visual inspection. The four students, who showed performance increases in all phases, had substantial to high PEM and PAND, as well as positive results in the Tau-U and the randomization test. In these students, the invention was at least partially effective. However, only in one case did the results reached statistical significance. The three students, who showed performance increases in the second A-phase, had low positive results in all measures, indicating very small performance gains across the study that cannot be clearly assigned to the intervention. The three students with no performance gains showed very low overlap indices as well as negative results in the randomization-based tests due to the baseline correction.

In two hierarchical piecewise regression models, the intervention effects regarding the acquisition of new mathematical knowledge and the maintenance of the new knowledge were analyzed across all ten students. To account for the heterogeneity of intervention effects that is suggested by the learning trajectories, random effects were added as recommended (Bell et al., 2019).

In this study, we distinguish between acquisition and the maintenance of new knowledge as two important effects of an intervention (McDougale et al., 2020). Acquisition refers to the performance increase after the start of the intervention. To investigate the intervention effect for the acquisition of new knowledge, the first A-phase is compared with the aggregated B-phases (see Table 3). Maintenance refers to preserving the newly learned knowledge after the end of the intervention (see Table 4).

#### 3.2.1. Acquisition

Regarding the acquisition of new knowledge, no statistically significant level or slope effect was found in the piecewise regression models. This means that during the intervention phases neither performance in the progression-based assessments nor the gain between them was significantly higher than in the baseline phase.

The standard deviations of the random effects were 20.359 for level and 3.725 for slope. This indicates that the students’ individual level and slope effects differed substantially, which corroborates the findings from the visual inspection. In sum, the individual parameters apparently cover a broad range, leading to non-significant overall intervention effects at the group level, but illustrating the heterogeneity in individual performance gains.

#### 3.2.2. Maintenance

Regarding the maintenance of newly acquired knowledge, the hierarchical piecewise regression model shows no significant level or slope effect. This suggests that the performance in the progression-based assessment and the gains between the measurement time points did not differ—but more importantly, did not decrease—when the intervention was paused.

Standard deviations of the random-slope effects in the maintenance model were similar to the ones of the acquisition model. Again, standard deviations of level (12.178) and slope (6.710) random effects were high in relation to the parameters of the fixed effects. Thus, it can be assumed that the individual maintenance of new knowledge was very divergent for different students, which might be disguised in the fixed effects.

### 3.3. Moderation Effects of Intelligence and Prior Knowledge for Intervention Effects (Research Question 2)

The visual analysis and the high standard deviations of the random effects suggest that the (lack of) intervention effects do not hold for all students equally. For this reason, moderator analyses were employed to investigate the influence of intelligence and prior knowledge on the effectiveness of the intervention.

Besides trend, level and slope effects, which address the effectiveness of the intervention in general, intelligence and prior knowledge as predictors as well as moderators (in form of interactions) for level and slope effects were added to the models. For ease of interpretation of the regression weights, both variables were z-standardized based on the current sample.

#### 3.3.1. Acquisition

To investigate the influence of intelligence and prior knowledge on the acquisition of new knowledge, moderator variables were added (see Table 5). As in the models focusing on intervention effects (Section 3.1), no significant intervention effect was found in the moderation model regarding acquisition. The only significant predictor for mathematical performance in the progression-based assessment was the interaction of intelligence and the slope effect. As the interaction coefficient was negative, higher intelligence was associated with a smaller increase in mathematic performance.

The random effects indicated substantial variation in the effects. The standard deviation for the level effect was 22.758 and for the slope effect was 4.710. Thus, whereas some students increased the achievement substantially, other students did not benefit, leading to an overall non-significant fixed effect.

#### 3.3.2. Maintenance

To test the moderation of the maintenance of possible intervention effects after the end of the first intervention phase, moderator variables were added to the maintenance model. Like the first model, this model revealed no significant level or slope effects of the second A phase (see Table 6). In this model, no significant effects or moderations by intelligence or prior knowledge were found.

The random effects indicated less variation, meaning that the maintenance effects remained comparable between students. This means that it is likely that there are small to no differences in performance level and slope between the intervention phase (B1) and the maintenance phase (A2). Thus, differential performance losses during the pause of the intervention are unlikely.

## 4. Discussion

This study aimed at investigating the effects of a computer-based mathematics intervention on math performance in students with different intelligence and prior knowledge and the way intelligence and prior knowledge might moderate intervention effects. Two possible intervention effects were focused: the immediate acquisition of new knowledge during the intervention and the maintenance of new knowledge directly after the end of the intervention.

### 4.1. Effectiveness of the Intervention (RQ1)

With regard to the first research question, we found that a general effectiveness of the intervention for all students with low mathematics performance is not supported by the results. The visual analysis and the standard deviations of the random effects of the hierarchical piecewise regression models revealed that the effectiveness of the intervention varied considerably between students. Whereas some students showed substantial performance gains in visual analysis and descriptive statistics, other students’ performance stagnated. A possible explanation for non-significant effects in the piecewise regression models could be—apart from the sample size—that the interindividual differences in the effectiveness between the students led to a generally not significant intervention effect that still might be substantial for some students but that cannot be generalized.

Whereas the hierarchical piecewise regression models did not reveal statistically significant (positive) intervention effects in the acquisition of new knowledge, significant (negative) effects after the end of the intervention (maintenance) were also not found. Thus, performance in the progression-based assessment did not drop significantly after the end of the intervention. This finding is supported by the means during the second A-phase.

While four students showed substantial performance gains during the intervention, more than half of the sample hardly responded to the intervention. One reason might be that Meister Cody mostly covers basic numerical skills that especially more intelligent students developed on their own. Thus, only students with very low cognitive capacities might benefit from a training with Meister Cody. Another reason for varying intervention effects might be the skills covered by the learning progression monitoring (CODY-LM) and the intervention (Meister Cody). Whereas the CODY-LM assessed addition, subtraction, and number comparison, Meister Cody trained these skills, but also number line estimation, early numeracy, and counting. As the intervention is supposed to be adaptive to the students’ individual profiles of an initial assessment, the frequency of the exercises provided during the intervention sessions varies. Thus, in some cases the learning progression monitoring might not match the intervention contents well enough to effectively measure progress. On the other hand, in some cases, the intervention might have focused intensively on the skills covered by the progression monitoring, leading to training-to-the-test effects. This is particularly striking, because the learning progression monitoring is part of the intervention program. In this study, the intervention (Meister Cody) and the aligned tests (CODY-M 2–4, CODY-LM) were used as intended and designed (Kuhn et al., 2018; Schwenk et al., 2017).

### 4.2. Moderation of Intervention Effects (RQ2)

With regard to the second research question, only one interaction was statistically significant in the hierarchical piecewise regression models. Intelligence negatively moderated the slopes of the performance gains when acquiring new knowledge. Thus, the higher the IQ of the students, the less they learned during the intervention. This result is not in line with Hassler Hallstedt et al. (2018), as students with lower IQ scores benefited more from the intervention. A possible reason for the different findings could be that the intervention that Hassler Hallstedt et al. (2018) trained mathematical contents (e.g., strategies) which more intelligent students are more likely to develop on their own. Thus, the intervention helps students without basic mathematical skills to catch up with their more-gifted peers. However, more-gifted students, who are likely to have already developed substantial parts of the contents of the intervention, would then benefit less from the intervention. It should also be noted that the mean of the IQ in our sample of students with mathematics difficulties was still below average when compared to the whole population as given in T-values. Summarizing the moderation effects, Meister Cody seems to be more effective for students who can be characterized by comparably high prior knowledge and rather low intelligence scores.

Prior knowledge, on the other hand, did not significantly moderate the effectiveness of the intervention in this study. However, all students who showed continuous performance gains over the whole time had comparably high scores in the prior knowledge assessment. Yet, this was not consistent as other students with similar prior knowledge benefited less from the intervention. One could argue that prior knowledge is thus a necessary, but not sufficient prerequisite for the effectiveness of a mathematics intervention.

Moderation analyses were included to explore potential differential effects of the intervention depending on intelligence. Given the limited number of observations inherent in single-case designs, particularly the small number of individuals, these findings are interpreted with appropriate caution. In particular, statistical inference of the results (e.g., the hierarchical piecewise regression models) requires careful interpretation. Both wrong positive (found interaction of slope and intelligence regarding acquisition) as well as wrong negative (non-significant interactions with prior knowledge) results are likely in the given sample. Thus, interaction effects in this context may inform future, more highly powered investigations.

It has to be mentioned that the two investigated individual characteristics—intelligence and prior knowledge—usually are correlated. For example, Kucian (2021) reports moderate-to-high correlations between intelligence and numeracy in kindergarten (*r* = 0.57) and first grade (*r* = 0.56). In addition, there is evidence that the correlation between general cognitive resources such as intelligence and domain-specific skills such as arithmetic performance is higher in low-performing students than in students with average performance or above (Tucker-Drob, 2009). In the hierarchical piecewise regression models, intelligence and prior knowledge mostly affect the intervention effects in the same direction. This indicates that the moderator effects of intelligence and prior knowledge are generally similar. Thus, the divergent moderations of intelligence and prior knowledge in the acquisition model (see Table 5) are of particular interest.

### 4.3. Limitations and Strengths

Although the current study gives valuable insights in the effectiveness of a computer-based mathematics intervention and the role of intelligence in this regard, several limitations have to be highlighted.

First, the heterogeneity of the intervention effects makes it hard to draw general conclusions. As the intervention affected the individual students’ performance differentially, finding common characteristics that moderate the effectiveness is difficult. Against the background of an exponentially growing number of control group intervention studies in mathematics (Reynvoet et al., 2021), the heterogenous responsivity of the students in this study emphasizes the need for investigating the effectiveness of an intervention not only on a group level (e.g., ANOVA of group means), but also on an individual level. This includes an analysis of reasons for differential effectiveness of an intervention.

Second, since this study is based on a single-case design involving students with low mathematical performance, the findings are hard to generalize for all students. The study covered a sample size of ten students. This sample size is above average for single-case design research (Shadish & Sullivan, 2011). Thus, the results provide relevant insights regarding the effects of a computer-based mathematics intervention and its moderation by intelligence and prior knowledge. However, a sample of this size is not representative for all students. Thus, replication studies are necessary to support (or restrain) the findings of this study. As the sample selection was intentionally based on the inclusion criterion of low math achievement, the intervention effects of this study and the moderator effect of intelligence only applies to this specific group of students. The selection of students is appropriate given that students with low mathematics performance are the target group for the intervention. However, the selected sample also limited the variance in intelligence. As a consequence, the sample included no students with above-average intelligence, which might interfere differently with a computer-based mathematics intervention. In particular, the moderation of the intervention effect by intelligence might not hold for students with above-average intelligence.

Third, only cognitive moderators of the intervention effects were investigated in this study. Research highlights the importance of other cognitive resources such as working memory or executive functions for mathematical learning (e.g., Peng et al., 2016). Although the individual characteristics of intelligence, working memory, and executive functions are closely related, a differential investigation might give valuable insights in the cognitive moderators of mathematical intervention effects. Besides cognitive characteristics, affective characteristics, such as motivation or mathematics anxiety might moderate the effectiveness of a mathematics intervention (Gaspard et al., 2023; Herzog et al., 2024). Moreover, there are behavior difficulties that occur frequently with mathematics difficulties, such as attention deficit/hyperactivity disorder (ADHD) or depression (Herzog & Casale, 2024; Visser et al., 2020). Given the substantial comorbidity rates between mathematics difficulties and behavior difficulties, the influence of behavior difficulties on the effectiveness of an intervention requires empirical investigation.

Fourth, the sample also showed substantial intra-individual variance regarding performance across the measurement time points. In the total sample, no significant trend effect was found. However, this led to positive or negative trends during the A1-phase in some cases. The intra-individual variance in the progression-based measurement might partially be explained by variance in mathematics performance—all students were low performing, but ranged from borderline to severe difficulties—and intelligence, which was inevitable against the background of the intention of the study.

In summary, the study provides valuable insight into the effects of prior knowledge and intelligence on the effectiveness of a computer-based mathematics intervention aiming at mathematics performance. The results highlight the role of intelligence for mathematics interventions that are not guided by teachers, but provide standardized learning opportunities that are only adapted regarding complexity (e.g., number range, carries). One interpretation might be that especially cognitive-capable students might miss metacognitive and interconnecting elements of mathematics teaching that link the learning goals of the different tasks and aim at a deeper understanding of arithmetic. Students with lower cognitive resources, however, might benefit more from interventions targeting procedures and fact retrieval (Moser Opitz et al., 2017). Further research is needed to address this hypothesis.

## Figures and Tables

**Table 1:** Overview of the participants of the study (see also Herzog & Casale, 2024).

Student ^1^	Age	Grade	Gender	HRT ^a^	CODY-M 2–4 ^a^	CFT ^a,2^	Training Sessions
Anton	10; 3	2	M	40	50	45	12
Bert	11; 2	2	M	23.5	29	45	11
Carl	7; 10	2	M	31	45	41	12
David	7; 10	2	M	28	24	30	12
Eric	7; 3	2	M	31	51	43	12
Fanny	7; 1	2	F	25	41	35	12
Gina	11; 3	4	F	21	55	46	10
Harold	10; 6	4	M	43	48	39	11
Ines	11; 5	4	F	38	35	39	10
Jenny	11; 10	4	F	35	46	30	10

Note: F = female; M = male; ^a^ T-values; ^1^ Pseudonyms; ^2^ CFT 1-R for students in Grade 2 and CFT 20-R for students in Grade 4.

**Table 2:** Descriptive statistics of the four phases of the intervention (see also Herzog & Casale, 2024).

	A1		B1		A1		B2						
	M (SD)	Md	M (SD)	Md	M (SD)	Md	M (SD)	Md	PEM	PAND	Tau-U	Hedge’s *g*	Randomization Test
Anton	139.75 (56.85)	160.0	141.83 (22.86)	138.5	145.83 (23.53)	140.0	144.00 (15.38)	149.0	50	45	0.01	−0.02	−0.48
Bert	62.25 (13.50)	63.0	57.50 (16.16)	61.0	74.33 (13.57)	76.5	69.20 (25.82)	62.0	18	43	−0.50	−036	−6.68
Carl	19.00 (23.41)	8.0	21.33 (19.10)	14.0	61.00 (18.65)	66.0	75.83 (22.71)	73.0	58	55	−0.11	0.13	4.38
David	10.83 (9.99)	8.5	15.33 (23.53)	6.0	17.50 (8.36)	21.0	17.67 (15.63)	16.5	33	50	−0.10	0.15	2.33
Eric	35.17 (11.16)	33.0	54.50 16.39)	52.5	79.40 (29.05)	68.0	92.67 (9.91)	91.5	75	57	0.10	0.65	18.31
Fanny	38.38 (13.75)	41.0	57.50 (30.98)	57.0	112.33 (10.52)	109.0	99.83 (14.08)	102.0	75	54	−0.11	0.22	8.60
Gina	85.75 (18.82)	85.5	127.00 (19.56)	118.0	111.20 (13.24)	105.0	129.50 (19.86)	131.0	100 ***	80 *	0.36 *	1.41	28.47
Harold	118.50 (27.76)	120.5	123.40 (15.47)	121.0	152.00 (33.53)	146.0	130.60 (22.66)	134.0	40	52	−0.11	−0.23	−6.73
Ines	149.62 (32.38)	146.0	164.00 (17.71)	156.0	199.00 (12.52)	197.5	176.40 (19.18)	171.0	30	36	−0.16	0.13	4.12
Jenny	106.00 (51.11)	115.0	138.20 (41.90)	13.0	187.50 (12.71)	188.5	204.00 (53.30)	220.00	60	62	−0.04	0.59	35.46

Note: A1 = first A-phase; B1 = first B-phase; A2 = second A-phase; B2 = second B-phase; M = mean; SD = standard deviation; Md = median; PEM = percentage exceeding the median; PAND = percentage of all non-overlapping data; Tau-U was specified as baseline corrected Kendall’s Tau; randomization test was specified as mean difference B vs. A; * *p* < .05; *** *p* < .001.

**Table 3:** Results of the hierarchical piecewise regression model regarding acquisition for intervention effects.

**Fixed Effects**	**B**	**SE**	**t**	***p***
Intercept	60.790	15.034	4.044	<.001
Trend	4.403	2.573	1.711	.089
Level	−11.575	9.524	−1.215	.226
Slope	−0.204	2.293	−0.089	.929
**Random Effects**	**SD**			
Intercept	42.387			
Trend	5.666			
Level	20.359			
Slope	3.725			
Residual	22.696			

Note: B = unstandardized model parameter; SE = standard error; t = result of the *t*-Test for significance; *p* = significance level; SD = standard deviation of the random-slope parameters.

**Table 4:** Results of the hierarchical piecewise regression model regarding maintenance for intervention effects.

**Fixed Effects**	**B**	**SE**	**t**	***p***
Intercept	80.348	16.906	4.753	<.001
Trend	3.057	2.560	1.194	.235
Level	9.494	8.701	1.091	.278
Slope	−0.559	3.251	−0.172	.864
**Random Effects**	**SD**			
Intercept	48.900			
Trend	5.982			
Level	12.178			
Slope	6.710			
Residual	19.563			

Note: B = unstandardized model parameter; SE = standard error; t = result of the *t*-Test for significance; *p* = significance level; SD = standard deviation of the random-slope parameters.

**Table 5:** Results of the hierarchical piecewise regression model regarding acquisition for moderators.

**Fixed Effects**	**B**	**SE**	**t**	***p***
Intercept	59.129	14.488	4.081	<.001
Trend	5.065	2.852	1.776	.078
Level	−12.426	10.201	−1.218	.225
Slope	−0.881	2.564	−0.344	.731
Intelligence	12.675	16.008	0.792	.454
Prior knowledge	7.078	15.986	0.433	.671
Level × Intelligence	7.517	7.436	1.011	.314
Level × Prior knowledge	3.406	7.394	0.461	.646
Slope × Intelligence	−2.780	1.248	−2.227	.027
Slope × Prior knowledge	1.705	1.235	1.380	.170
**Random Effects**	**SD**			
Intercept	39.437			
Trend	6.437			
Level	22.758			
Slope	4.710			
Residual	22.361			

Note: B = unstandardized model parameter; SE = standard error; t = result of the *t*-Test for significance; *p* = significance level; SD = standard deviation of the random-slope parameters.

**Table 6:** Results of the hierarchical piecewise regression model regarding maintenance for moderators.

**Fixed Effects**	**B**	**SE**	**t**	***p***
Intercept	80.294	15.512	5.176	<.001
Trend	3.047	2.565	1.188	.238
Level	9.470	8.205	1.187	.238
Slope	−0.704	3.422	−0.206	.837
Intelligence	4.673	17.725	0.264	.800
Prior knowledge	20.994	17.716	1.185	.275
Level × Intelligence	−11.089	9.476	−1.170	.245
Level × Prior knowledge	−7.168	9.377	−0.764	.447
Slope × Intelligence	0.751	2.196	0.342	.733
Slope × Prior knowledge	3.235	2.093	1.546	.126
**Random Effects**	**SD**			
Intercept	42.828			
Trend	5.742			
Level	6.727			
Slope	7.086			
Residual	19.272			

Note: B = unstandardized model parameter; SE = standard error; t = result of the *t*-Test for significance; *p* = significance level; SD = standard deviation of the random-slope parameters.

## Data Availability

The data presented in this study are available on request from the corresponding author due to missing consent by participants to publish in repository.
